# Evaluation of clinical performance and survival rate of Straumann dental implants in Saudi Population based on cross-sectional study

**DOI:** 10.1038/s41598-021-89112-8

**Published:** 2021-05-04

**Authors:** Reham AL Jasser, Mohammed AlSarhan, Dalal Alotaibi, Saleh Aloraini, Pradeep Koppolu, Sebastiano Andreana

**Affiliations:** 1 Department of Periodontics and Community Dentistry, Dental College, King Saud University, Riyadh, Kingdom of Saudi Arabia; 2 Department of Preventive Dental Sciences, College of Dentistry, Dar Al Uloom University, Riyadh, Kingdom of Saudi Arabia; 3 Department of Restorative Dentistry, State University of New York at Buffalo School of Dental Medicine, Buffalo, New York USA

**Keywords:** Peri-implantitis, Risk factors

## Abstract

Risk indicators of peri-implantitis is still contradictory and somehow unclear in present literature therefore efforts should be done for better understanding of the exact etiology of peri-implant disease progression. The present study aimed to assess risk indicators associated with peri-implantitis by observing the changes in several periodontal parameters after implant placement. This cross-sectional study included 213 female and 271 male patients aged 26–87 years, who received 484 titanium implants (Straumann, Switzerland) at King Saud University’s Dental College, Saudi Arabia. Patients were called for dental visits. During these visits; full clinical and radiographic assessment of implants were done. The periodontal pocket depth (PPD) was greater around implants placed at grafted sites than non-grafted sites and around bone-level implants than tissue-level implants. The plaque index (PI) was associated with poor oral hygiene. There was a strong association between graft (yes/no) and bleeding on probing (BOP). Patients with good oral hygiene showed high radiographic bone stability. Keratinized tissue width < 2 mm was associated with a higher PPD, higher PI, higher BOP, more edematous gingiva, and more exposed implant threads on radiography. In patients receiving implants, poor oral hygiene status and inadequate keratinized tissue level can be proposed as risk indicators for developing periimplantitis due to strong association found between them and developments of peri-implantitis.

## Introduction

Dental implants are becoming more globally preferred procedure for supporting missing teeth in daily clinical practice due to their high overall success rate^1^. However, specific risk factors leading to implant failures need to be considered prior to their use^2^. The success of dental implants as a replacement for missing teeth is lowered by the complications of peri-implant mucositis and periimplantitis^3^. According to new scheme of periodontal and implant diseases classification^4^, peri-implant mucositis is defined as the reversible inflammation of soft tissue surrounding dental implants, whereas peri-implantitis is described as the irreversible form of the inflammatory process due to degeneration of connective tissue between bone and osseointegrated oral implants usually followed by bone resorption^2,4^. The imbalance between the bacterial challenge and host response at the soft tissue–implant interface triggers this inflammatory process, predicted to be different from those observed around natural teeth in periodontal disease^2^. Such diseases are primarily caused by the colonization of different pathogenic bacteria on these implants’ surfaces in forms of bacterial biofilms.

As more dental implants were in place, more long-term complications were observed upon different implant placement scenarios. Several theories and thoughts were discussed through dental implant literature by several case reports, series.

Efforts have been made to understand the primary etiological causes of these diseases. Although speculations pointed that basic principles of biofilm’s formation are similar to periodontal and implant disease due to their presence in similar oral environment^5,6^. Overall it is crucial to understand that pathogenic microbiota still remains the main etiological factor of developing periimplantitis. Furthermore, several studies have shown that microbiota may be different due to the presence of inert materials mainly titanium in the ecological setting^6,7^. Another factor which plays major role in microbial changes is the difference of blood supply as well as cytokines surrounding the dental implant comparable to natural tooth^8^.

Furthermore, it is essential to identify peri-implantits. Additional risk factors and indicators that can cause more destructions and failures of implants in order to understand the progressive pathway of peri-implant disease^9–12^. Several risk factors were proposed based on longitudinal studies and risk indicators based on other cross-sectional studies have been debated to be additional influencers on peri-implantitis. These include and not limited to; risk factors such as smoking, diabetes and risk indicators such as osteoporosis and local factors including restorative part mishaps and presence of excessive cement^13,14^. Furthermore, this cross-sectional study aimed to assess risk indicators associated with different stages of peri-implantitis at implant-based analysis by observing changes in several periodontal parameters over a period of three years post- implant placement in Saudi Arabia.

## Materials and methods

### Study design and participants

The study was conducted on patients receiving conventional length (> 6 mm), non-turned, 2- and 3-piece titanium implants* (Straumann, Switzerland) in King Saud University’s Dental College, Riyadh, Saudi Arabia during the period 2015 to 2018. The patients were randomly included and scheduled for dental appointments; all clinical parameters were collected from June 2018 to September 2019 during regular implant maintenance visits. Furthermore, all clinical measurements were taken by a single blinded trained and calibrated investigator to exclude possible operator-dependent bias.

### Inclusion criteria

In accordance with Konstantinidis et al.^15^ and Schwarz et al.^16^ for patient selection, sample of the study consisted of systemically healthy partially edentulous patients with one or more missing teeth being replaced by single crown implant-supported restoration with a minimum period of 6 months of functional occlusal loading during the appointment for evaluation. Implant surgical operations and prosthetic restorations were all performed in the same institute.

### Exclusion criteria

The following exclusion criteria were defined: (1) any uncontrolled systemic diseases that could influence the implant therapy outcome (e.g., diabetes [HbA1c > 7], osteoporosis); (2) Smoking; (3) pregnancy or breastfeeding in women; (4) intake of medications having an effect on bone turnover and mucosal healing (i.e., steroids, antiresorptive therapy); (5) antibiotic use for a medical or dental reason within the 2 months prior to the examination; (6) any restorations that did not allow for the calculation of periodontal pocket depths(PPD); (7) inability or refusal to sign the informed consent form and (8) the absence of baseline radiographs taken at the time of placement of the implant or final crown^15,16^.

### Anamnestic data and implant site characteristics

The following study variables were assessed at the time of final examination: (1) age (2) gender. The following implant site characteristics were considered: (1), implant type (bone level vs. tissue level), (2) implant size, (3) implant location (maxillary vs. mandibular arch, (4) grafting the area (yes/no), (5) graft type (Autograft vs. Allograft vs. xenograft) and (6) type of allograft in terms of particulate (Cortical vs. Cancellous vs. Mixed) * (ACE Surgical Supply Co., Brockton, MA, USA).

### Clinical measurements

The clinical parameters were assessed for each implant. Periodontal probing depth (PPD) was measured using plastic probe (11 Colorvue Probe, Hu-Friedy) by inserting the probe within sulcus area with gentle pressure (less than 0.25Ncm) around the neck of an implant at three points buccal and three points lingual to the implant having the probe placed parallel to the crown of the implant at mid buccal and mid lingual points and 10 degree tilted inward at the proximal points to the nearest mm^17^. Bleeding on probing (BOP) was assessed by either the presence (+) or absence (−) of bleeding at the site of probing immediately after periodontal pocket depth measurement^2^. Plaque index (PI) was assessed by either the presence (+) or absence (−) of Plaque on four surfaces (mesial, distal, palatal and buccal) of crown after placing disclosing agent* (Hu-Friedy Mfg. Co., Chicago, IL, USA). The measurements of PPD, BOP, and PI were performed at six aspects per implant: mesio-buccal, mid-buccal, disto-buccal, and respective lingual/palatal sites^3^. Gingival color and consistency were evaluated via direct visual assessment, i.e., visibility of the periodontal probe , Gingival changes were determined by assessing redness area visible between the level of the inter-proximal papillae and the gingival margin.as redness is an indication for inflammation color pink is indication or gingival health^4^.

Keratinized tissue width (KTW) of presence or absence of (≥ 2 mm) was assessed from the peri-implant marginal mucosa to the muco-gingival junction (MGJ) at the buccal and lingual side of each implant. KTW was measured round the implant and calculated by the mean of four margins (buccal, lingual, mesial, and distal) by the aid of the periodontal probe. The former was measured from the peri-implant marginal mucosa to the muco-gingival junction (MGJ) at the buccal and lingual marginal portion of the implant’s mucosa^6^. Finally, standardized periapical radiographs were taken at the time of the clinical examination with the long cone paralleling technique and film holders (Rinn XCP, Dentsply Corporate, PA, USA) and compared with a baseline radiograph taken at the time of prosthesis installation for bone level confirmation at the same facility^14^. Particularly, the radiographs were scanned to obtain standardized digital images with a resolution of 1200 dpi. These images were imported and analyzed using specialized computer software (Image J v 1.49, Research Services Branch, National Institute of Health, Bethesda, MD, USA). The calibration of the pixel/mm ratio was performed using the length of the implant as a fixed reference point to compensate for potential radiographic distortion. For the assessment of bone loss, the radiographic distance between the implant shoulder level and the most coronal bone-to-implant contact level was measured mesially and distally, parallel with the long axis of the implant. The same-blinded examiner performed all radiographic measurements^18^.

### Data analysis

Data were analyzed using SPSS 24.0 version statistical software (IBM Inc., Chicago, USA). Descriptive statistics (mean, standard deviation, frequencies and percentages) were used to describe the quantitative outcome variable (PPD, KTW), categorical outcome variables including (PI), (BOP), oral hygiene, Gingival color, consistency, and radiographic bone loss and other categorical study variables (age groups, gender, implant type, implant size, implant location, graft vs. none graft as well as graft type). In addition, oral hygiene status and KTW were evaluated. Student’s t-test for independent samples and one-way analysis of variance were used to compare the mean values of PPD in relation to the categorical study variables. Pearson’s Chi-square test was used to assess the association between the categorical study and outcome variables. Karl Pearson’s correlation coefficient was used to quantify the relationship between attached gingiva values and PPD scores. A *p*-value of < 0.05 was used to report the statistical significance of results.

### Ethical approval

The present cross-sectional study was carried out in accordance with the Helsinki Declaration of 1975, as revised in 2013. The protocol used in this study was approved by the Institutional Committee of Research Ethics at King Saud University, Riyadh, Saudi Arabia (87,563). Each patient was given a detailed description of the procedure, and informed consent was obtained prior to participation in the study.

## Results

From 2045 screened patients’ records, 213(48.1%) female patients and 271 (51.2%) male patients aged 26–87 years (mean, 60 ± 8.6 years) were selected due to sufficient clinical data. Among these selected patients, a total of 484 dental implants were inserted, 251 (56%) into female patients and 233 (49%) into male patients. According to definition given, out of 484 implants, 201 (42%) presented with BOP on more than one surface area around the implant and were therefore diagnosed with peri-implant mucositis. Further analysis using means of periapical X-rays, 115 (23.76%) revealed peri-implant marginal bone loss, and therefore, out of all implants included in present study, 42% sites showed peri-implant-mucositis while 23.76% showed peri-implantitis.

### Periodontal pocket depth (PPD)

**Figure 1:**
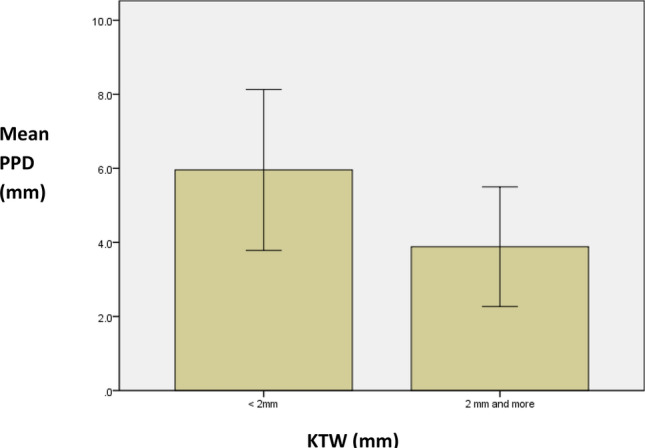
Mean of periodontal pocket depth (PPD) in mm based on keratinized tissue width (KTW) around implants.

The mean PPD was deeper around implants placed in grafted sites versus non-grafted sites (6.82 ± 2.27 mm) versus (4.60 ± 2.0 mm), this difference was statistically significant *p* < 0.001. Additionally, It showed that PPD was statistically significantly deeper around bone level implants in comparison to tissue level (6.70 ± 2.1 mm) versus (4.61 ± 2.0 mm), *p* < 0.001). Furthermore, PPD was also shown to be statistically significant in patients exhibiting poor oral hygiene in comparison to individuals either with fair or good oral hygiene (13.39 ± 1.31 mm vs. 6.35 ± 1.87 mm) versus (4.15 ± 1.22 mm) *p* < 0.001. Finally PPD showed statistical significant association with the presence of (≥ 2 mm) KTW, higher PPD was associated with KTW less than 2 mm compared to PPD in implants with KTW ≥ 2 mm, (5.96 ± 2.17 mm) and (3.88 ± 1.61 mm) respectively, *p* < 0.004) (Table 1) (Fig. 1). Overall, PPD values were not statistically different in relation to the following variables; age groups, gender, implant type, implant size, implant location, graft (yes/no), graft type and type of allograft. The comparison of mean values of PPD shows statistically significant difference in relation to oral hygiene status and attached gingiva. In regards to correlation, there was significant negative correlation between KTW and PD scores (r = − 0.461, *p* < 0.004). That is, as KTW values increased the PPD decreased.

**Table 1:** Comparison of mean values of PPD in relation to the study variables.

Study variables	PD-mean (SD)	F-value/*t*-value	*p*-value
**Age groups (in years) (n = 484)**			
≤ 40	4.64(2.0)	0.034	0.966
41–60	4.68(2.1)		
> 60	4.70(2.1)		
**Gender**			
Male	4.46(2.1)	1.969	0.050
Female	4.87(2.1)		
**Implant type**			
Bone level	6.70(2.1)	0.395	< 0.001**
Tissue level	4.61(2.0)		
**Implant size**			
3.3 × 8 mm	4.52(2.2)	0.338	0.917
3.3 × 10 mm	4.64(1.9)		
4.1 × 8 mm	4.55(2.1)		
4.1 × 10 mm	4.67(2.1)		
4.1 × 12 mm	4.44(2.1)		
4.8 × 8 mm	4.87(2.0)		
4.8 × 10 mm	5.03(2.4)		
**Implant location**			
Maxillary	4.84(2.2)	1.725	0.085
Mandibular	4.51(2.0)		
**Graft**			
Yes	6.82(2.27)		
No	4.60(2.0)	1.095	< 0.001**
**Graft type**			
Auto graft	6.75(2.32)		
Allograft	4.50(1.22)		
Xeno graft	4.68(2.1)	1.765	0.274
**Among allograft**			
Cancellous	5.47(2.24)		
Cortical	5.06(2.82)		
Mixed	4.35(2.14)		
**Oral hygiene status**			
Poor	13.39(1.31)		
Fair	6.35(1.87)	2.173	< 0.001**
Good	4.15(1.22)		
KTW*			
< 2 mm	5.96(2.17)		
2 mm and more	3.88(1.61)	212.612	< 0.004**

*Keratinized tissue width.

**Statistically significant.

### Plaque index (PI)

**Figure 2:**
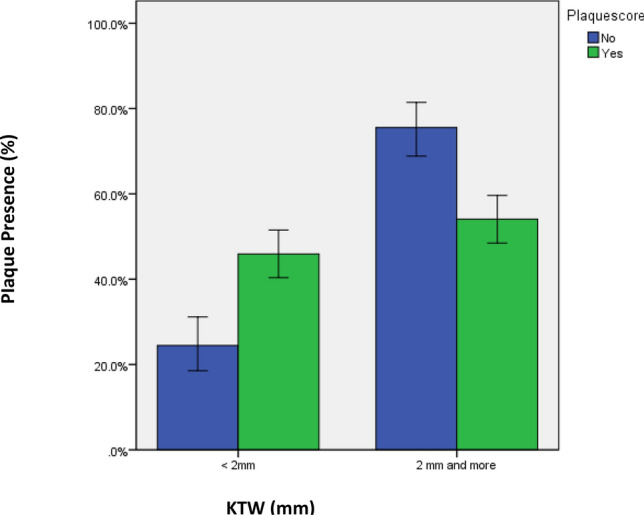
Percentage of plaque presence (yes vs. no) based on keratinized tissue width (KTW) around implants.

The distribution of PI showed statistically significant association with oral hygiene status and KTW. A highly statistically significant association was shown between presence of plaque and inferior state of oral hygiene status (poor, fair and good) (182 ± 49.8%) versus (170 ± 82.9%) versus 7 ± 36.8%); *p* < 0.0001). A higher PI was associated with KTW less than 2 mm compared to PI in implants with KTW ≥ 2 mm, (165 ± 76.5%) and (140 ± 55.4%) respectively, *p* < 0.003 (Table 2) (Fig. 2).

**Table 2:** Association between PI and other study variables.

Study variables	PI-Frequency (%)		Χ^2^-value	*p*-value
	Yes	No		
**Age groups (in years) (305;176)**				
≤ 40	84(63.2)	49(36.8)	0.019	0.990
41–60	125(63.8)	71(36.2)		
> 60	96(63.2)	56(36.8)		
**Gender (261;150)**				
Male	134(63.2)	78(36.8)	0.017	0.898
Female	127(63.8)	72(36.2)		
**Implant type (299;176)**				
Bone level	221(64.8)	120(35.2)	1.797	0.180
Tissue level	78(58.2)	56(41.8)		
**Implant size (323;151)**				
3.3 × 8 mm	18(66.7)	9(33.3)	7.951	0.242
3.3 × 10 mm	49(69)	22(31)		
4.1 × 8 mm	5(55.6)	4(44.4)		
4.1 × 10 mm	161(86.6)	92(36.4)		
4.1 × 12 mm	23(48.9)	24(51.1)		
4.8 × 8 mm	24(60)	16(40)		
4.8 × 10 mm	25(75.8)	8(24.2)		
**Implant location (305;176)**				
Upper	157(64.9)	85(35.1)	0.451	0.502
Lower	148(61.9)	91(38.1)		
Graft (305;176)				
Yes	112(65.5)	59(34.5)	0.498	0.480
No	193(62.3)	117(37.7)		
**Graft type (111;59)**				
Auto graft	92(66.2)	47(33.8)	0.363	0.834
Allograft	4(66.7)	2(33.3)		
Xeno graft	15(60)	19(40)		
**Among allograft (192;138)**				
Cancellous	21(65.6)	111(34.4)	4.503	0.342
Cortical	119(78.9)	4(21.1)		
Mixed	52(69.3)	23(30.7)		
**Oral hygiene status (359;176)**				
Poor	182(49.8)	12(63.2)		
Fair	170(82.9)	35(17.1)		
Good	7(36.8)	129(50.2)	59.936	< 0.0001**
**KTW (305;176)**				
< 2 mm	165(76.5)	43(23.5)		
2 mm and more	140(55.4)	133(44.6)	21.825	< 0.003**

*Keratinized tissue width.

**Statistically significant.

### Bleeding on probing (BOP)

The distribution of BOP across the study variables showed a highly statistically significant association between graft (yes/no) and BOP (83 ± 45.6%) versus (5 ± 55.6%), *p* < 0.001).

A statistically significant association with the type of graft done as subjects (62.8%) whose received autograft were having BOP when compared to other types of grafts (11 ± 26.8%) versus (2 ± 100%) versus (3 ± 33.3%), *p* < 0.001). A higher BOP was associated with KTW less than 2 mm compared to PI in implants with KTW ≥ 2 mm, (85 ± 52.1%) and (3 ± 42.4%) respectively, *p* < 0.0001 (Table 3).

**Table 3:** Association between BOP and other study variables.

Study variables	BOP-frequency (%)		Χ^2^-value	*p*-value
	Yes	No		
**Age groups (in years) (88;103)**				
≤ 40	27(46.6)	31(53.4)	3.730	0.155
41–60	39(53.4)	34(46.6)		
> 60	22(36.7)	38(63.3)		
**Gender (76;86)**				
Male	34(43)	45(57)	0.930	0.335
Female	42(50.6)	41(49.4)		
**Implant type (86;103)**				
Bone level	55(42)	76(58)	2.130	0.144
Tissue level	31(53.4)	27(46.6)		
**Implant size (87;124)**				
3.3 × 8 mm	3(25)	9(75)	8.955	0.176
3.3 × 10 mm	15(55.6)	12(44.4)		
4.1 × 8 mm	3(60)	2(40)		
4.1 × 10 mm	44(44.9)	54(55.1)		
4.1 × 12 mm	12(54.5)	10(45.5)		
4.8 × 8 mm	9(52.9)	8(47.1)		
4.8 × 10 mm	12(11.1)	8(88.9)		
**Implant location (88;103)**				
Upper	43(51.8)	40(48.2)		
Lower	45(41.7)	63(58.3)	0.342	0.559
**Graft (88;103)**				
Yes	83(45.6)	4(54.4)		
No	5(55.6)	99(44.4)	1.102	< 0.001**
**Graft type (16;103)**				
Autograft	11(62.8)	2(4.9)		
Allograft	2(100)	10(10)	55.152	< 0.0001**
Xeno graft	3(33.3)	91(65)		
**Among allograft (45;39)**				
Cancellous	40(40)	3(73.2)		
Cortical	3(75)	6(66.7)	6.736	0.009
Mixed	2(100)	30(100)		
**Oral hygiene status (88;103)**				
Poor	17(94.4)	1(5.6)	4.827	0.090
Fair	47(73.4)	17(26.6)		
Good	24(22)	85(78)		
KTW*(88;103)				
< 2 mm	85(52.1)	3(5.2)	61.624	< 0.0001**
2 mm and more	3(42.4)	100(57.6)		

*Keratinized tissue width.

**Statistically significant.

### Gingival color and consistency

**Figure 3:**
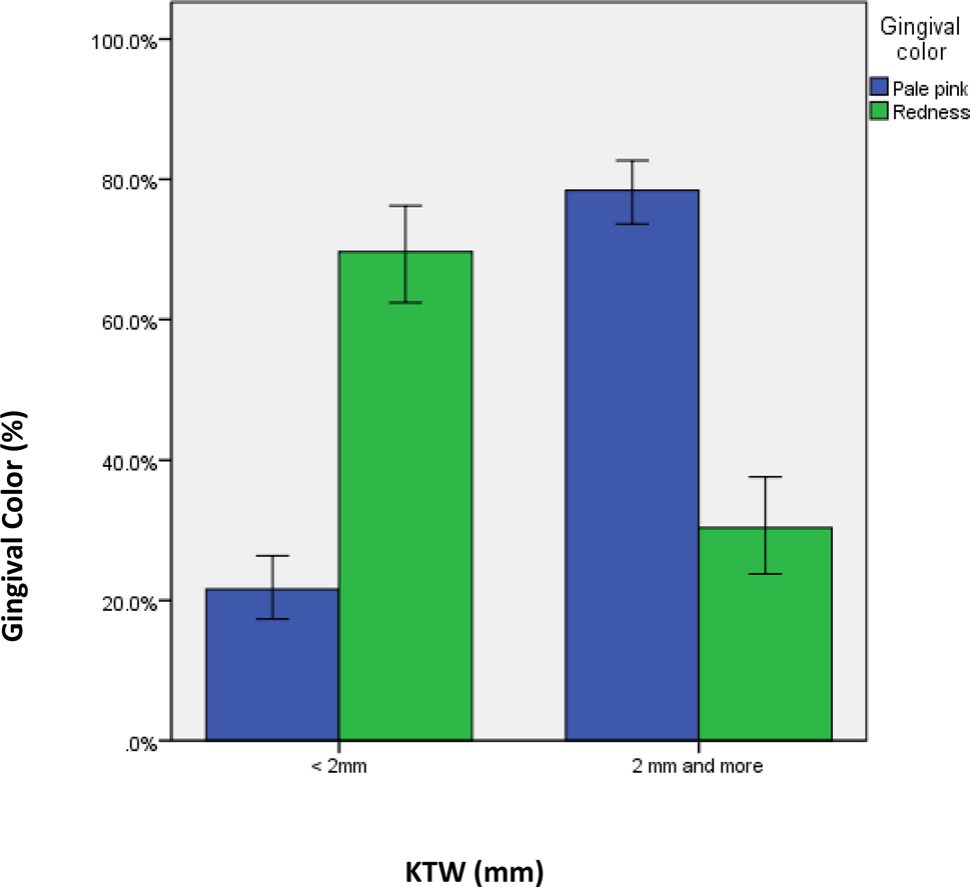
Percentage of gingival color (pale pink vs. redness) based on keratinized tissue width (KTW).

Lower implants had a significant higher proportion of pale pink gingival color as compared to upper implants (141 ± 57.8%) versus (174 ± 72.8%), *p* = 0.001) which was highly statistically significant. This significance was also shown when color was compared based on oral hygiene status (17 ± 85%) versus (56 ± 27.3%) versus (242 ± 93.8%), *p* < 0.0001) and KTW (247 ± 82.9%) versus (68 ± 36.8%), *p* < 0.0001) (Table 4) (Fig. 3).

**Table 4:** Association between Gingival color and other study variables.

Study variables	Gingival color-frequency (%)		Χ^2^-value	*p*-value
	Pale pink	Redness		
**Age groups (in years) (415;168)**				
≤ 40	88(66.2)	45(33.8)	0.090	0.956
41–60	129(65.2)	69(34.8)		
> 60	98(64.5)	54(35.5)		
**Gender (271;142)**				
Male	148(69.5)	65(30.5)	2.914	0.088
Female	123(61.5)	77(38.5)		
**Implant type (314;163)**				
Bone level	218(63.6)	125(36.4)	2.800	0.094
Tissue level	96(71.6)	38(63)		
**Implant size (326;168)**				
3.3 × 8 mm	17(63)	10(37)	4.586	0.598
3.3 × 10 mm	42(57.5)	31(42.5)		
4.1 × 8 mm	7(77.8)	2(22.2)		
4.1 × 10 mm	173(68.4)	80(31.6)		
4.1 × 12 mm	19(57.6)	14(42.4)		
4.8 × 8 mm	30(63.8)	17(36.2)		
4.8 × 10 mm	26(65)	14(35)		
**Implant location (315;168)**				
Upper	141(57.8)	103(42.2)	12.002	0.001**
Lower	174(72.8)	65(27.2)		
**Graft (315;168)**				
Yes	102(59.6)	69(40.4)	3.618	0.050**
No	213(68.3)	99(31.7)		
**Graft type (126;68)**				
Auto graft	85(61.2)	54(38.8)	1.855	0.396
Allograft	3(33.3)	4(66.7)		
Xeno graft	15(60)	10(40)		
**Among allograft (88;68)**				
BiossCancellous	18(56.2)	14(43.8)	6.748	0.150
Cortical	17(42.1)	11(57.9)		
Mixed	53(33.3)	16(47.1)		
**Oral hygiene status (315;168)**				
Poor	17(85)	3(15)		
Fair	56(27.3)	149(72.7)		
Good	242(93.8)	16(6.2)	226.170	< 0.0001**
**KTW (315;168)**				
< 2 mm	68(36.8)	117(63.2)		
2 mm and more	247(82.9)	51(17.1)	107.070	< 0.0001**

*Keratinized tissue width.

**Statistically significant.

**Figure 4:**
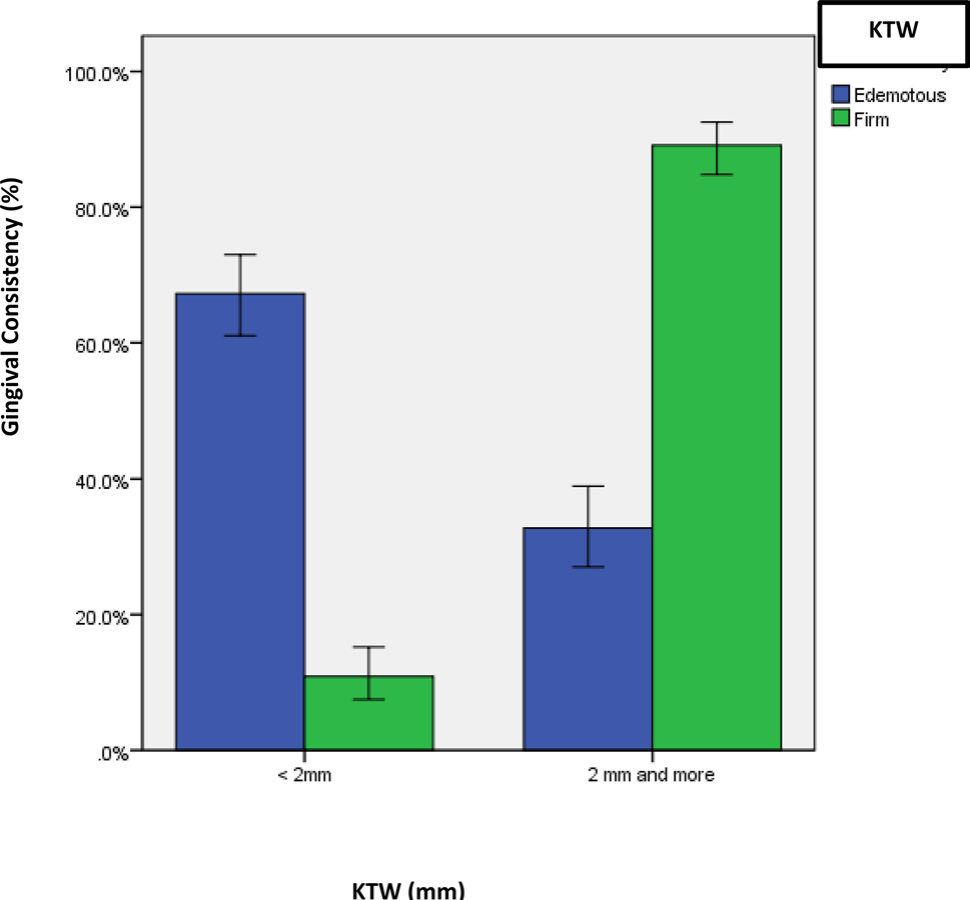
Percentage of gingival consistency (firm vs. edematous) based on amount of KTW.

**Figure 5:**
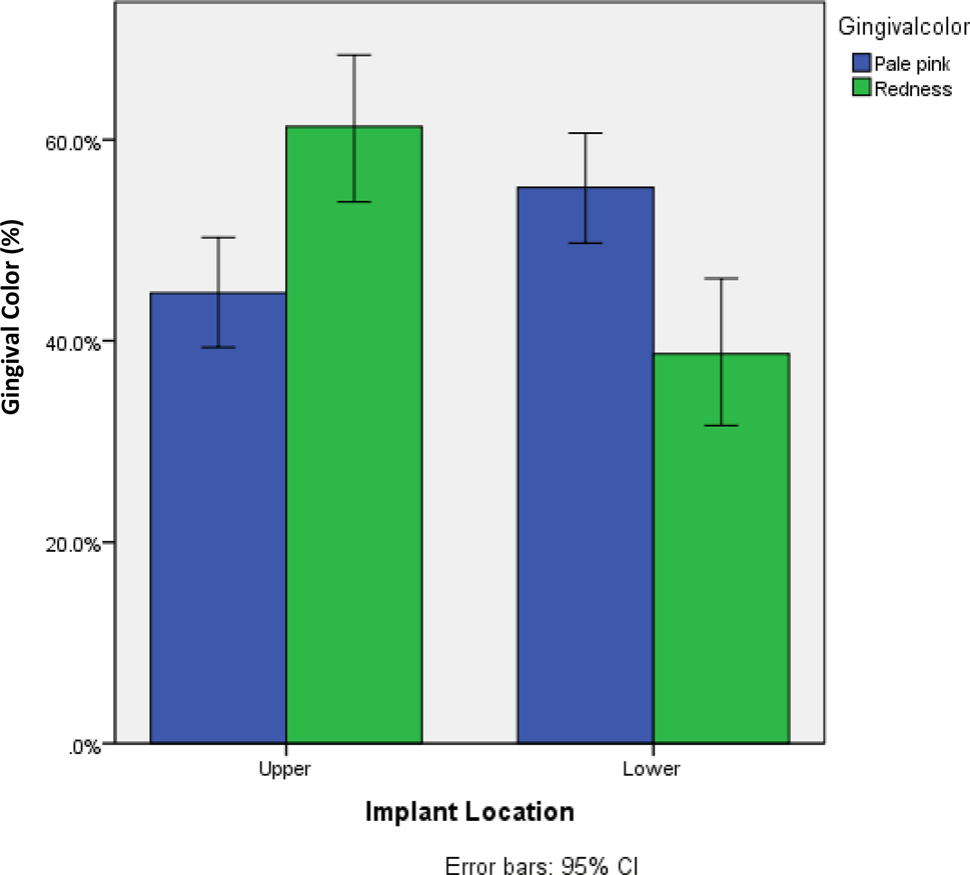
Percentage of gingival color (pale pink vs. redness) based on implant location (maxillary vs. mandibular).

As for gingival consistency, subjects received “Autograft” presented with more edematous soft tissue surrounding the implants in compared to other types of grafts (64 ± 56%) versus 6 ± 10%) versus (10 ± 40%), *p* = 0.026). Furthermore, among allografts, Cancellous type was highly statistically significantly associated with presence of edematous consistency (26 ± 43.8%) when compared to either cortical (13 ± 68.4%) or mixed type 16 ± 100%) (*p* = 0.001). Similarly, poor and fair categories of oral hygiene status had more edematous consistency when compared with good oral hygiene status indicating highly statistically significant association (162 ± 79%) versus 58 ± 22.5%) versus 15 ± 25%), *p* < 0.0001). Finally, presence of < 2 mm KTW presented with more edematous gingiva surrounding the implant when it was compared with subjected presented with (≥ 2 mm) keratinized tissue which was again highly statistically significant (158 ± 85.4%)versus 77 ± 25.8%), *p* < 0.0001) (Table 5) (Figs. 4, 5).

**Table 5:** Association between consistency and other study variables.

Study variables	Consistency		Χ^2^-value	*p*-value
	Edemotous	Firm		
**Age groups (in years) (235;248)**				
≤ 40	65(48.9)	68(51.1)	0.159	0.924
41–60	98(49.5)	100(50.5)		
> 60	72(47.4)	80(52.6)		
**Gender (199;214)**				
Male	94(44.1)	119(55.9)	2.893	0.089
Female	105(52.5)	95(47.5)		
**Implant type (230;247)**				
Bone level	161(46.9)	182(53.1)	0.800	0.371
Tissue level	69(51.5)	65(48.5)		
**Implant size (234;248)**				
3.3 × 8 mm	13(48.1)	14(51.9)	1.458	0.962
3.3 × 10 mm	34(46.6)	39(53.4)		
4.1 × 8 mm	4(44.4)	5(55.6)		
4.1 × 10 mm	123(48.6)	130(51.4)		
4.1 × 12 mm	24(51.1)	23(48.9)		
4.8 × 8 mm	22(55)	18(45)		
4.8 × 10 mm	14(42.4)	19(57.6)		
**Implant location (235;248)**				
Upper	124(50.8)	120(49.2)	0.926	0.336
Lower	111(46.4)	128(53.6)		
**Graft**				
Yes	81(47.4)	90(52.6)	0.175	0.676
No	154(49.4)	158(50.6)		
**Graft type (80;90)**				
Auto graft	64(56)	75(54)	7.308	0.026**
Allograft	6(10)	0(0)		
Xeno graft	10(40)	15(60)		
**Among allograft (55;93)**				
Cancellous	26(43.8)	18(56.3)	18.727	0.001**
Cortical	13(68.4)	26(31.6)		
Mixed	16(100)	49(65.3)		
**Oral hygiene status (235;248)**				
Poor	162(79)	5(25)	151.993	< 0.0001**
Fair	58(22.5)	43(21)		
Good	15(25)	200(77.5)		
**KTW (235;248)**				
< 2 mm	158(85.4)	27(14.6)	162.114	< 0.0001**
2 mm and more	77(25.8)	221(74.2)		

*Keratinized tissue width.

**Statistically significant.

### Radiographic findings

In relation to oral hygiene status, good oral hygiene group showed significant more radiographic bone stability relative to other groups (234 ± 90.7%) when compared to either fair (86 ± 42%) or poor (17 ± 85%), *p* < 0.001).

Bone loss exposing the threads was significantly associated with poor oral hygiene when compared with good oral hygiene group, 262 ± 5%) and 2 ± 12.7%) respectively (*p* < 0.001). Implants with KTW less than 2 mm had more exposed threads shown in the radiograph (246 ± 82.6%) compared to implants with KTW ≥ 2 mm 91 ± 49.2%), this difference was statistically significant *p* < 0.001 (Table 6).

**Table 6:** Association between radiographic findings and other study variables.

Study variables	Radiographic findings					Χ^2^-value	*p*-value
	No change	1 thread exposed	2 threads exposed	3 threads exposed	5 threads exposed or more		
**Age groups (in years)**							
≤ 40	97(72.9)	6(4.5)	8(6)	17(12.8)	5(3.8)	4.774	0.781
41–60	138(69.7)	7(3.5)	19(9.6)	21(10.6)	13(6.6)		
> 60	102(67.1)	4(2.6)	14(9.2)	21(13.8)	11(7.2)		
**Gender**							
Male	157(73.7)	6(2.8)	14(6.6)	26(12.2)	10(4.7)	5.743	0.219
Female	135(67.5)	10(5)	21(10.5)	19(9.5)	15(7.5)		
**Implant type**							
Bone level	236(68.8)	11(3.2)	31(9)	41(12)	24(7)	4.354	0.360
Tissue level	99(73.9)	5(3.7)	8(6)	18(13.4)	4(3)		
**Implant size**							
3.3 × 8 mm	18(66.7)	2(7.4)	2(7.4)	4(14.8)	1(3.7)	0.911	0.574
3.3 × 10 mm	53(72.6)	3(4.1)	7(9.6)	8(11)	2(2.7)		
4.1 × 8 mm	6(66.7)	1(11.1)	1(11.1)	1(11.1)	0		
4.1 × 10 mm	178(70.4)	7(2.8)	24(9.5)	28(11.1)	16(6.3)		
4.1 × 12 mm	22(66.7)	1(3)	0	6(18.2)	4(12.1)		
4.8 × 8 mm	32(68.1)	2(4.3)	5(10.6)	6(12.8)	2(4.3)		
4.8 × 10 mm	27(67.5)	1(2.5)	2(5)	6(15.0)	4(10)		
**Implant location**							
Upper	161(66)	8(3.3)	24(9.8)	35(14.3)	16(6.6)	4.231	0.923
Lower	176(73.6)	9(3.8)	17(7.1)	24(10)	13(5.4)		
**Graft**							
Yes	112(65.5)	8(4.7)	17(9.9)	22(12.9)	12(7)		
No	225(72.1)	9(2.9)	24(7.7)	37(11.9)	17(5.4)	223.699	0.625
**Graft type**							
Auto graft	91(65.5)	8(5.8)	11(7.9)	20(14.4)	9(6.5)		
Allograft	3(50)	0	1(16.7)	0	2(33.3)	2.906	0.574
Xeno graft	17(68)	0	5(20)	2(8)	1(4)		
**Among allograft**							
Cancellous	30(65.6)	1(3.1)	7(15.6)	9(26.3)	5(15.8)		
Cortical	9(52.9)	1(5.9)	4(17.6)	3(17.6)	8(33.3)		
Mixed	58(73.3)	6(8)	6(8)	5(6.7)	0(4.0)	0.911	0.910
**Oral hygiene status**							
Poor	17(85)	1(5)	1(5)	53(23)	262(5)		
Fair	86(42)	9(4.4)	30(14.6)	20(26.3)	2(12.7)		
Good	234(90.7)	10(2.7)	7(3.9)	5(1.9)	0	141.767	< 0.001*
**Attached gingiva**							
< 2 mm	91(49.2)	9(4.9)	28(15.1)	38(20.5)	19(10.3)		
2 mm and more	246(82.6)	8(2.7)	13(4.4)	21(7)	10(3.4)	61.456	< 0.001*

*Statistically significant.

## Discussion

Due to the advancements made in the field of Dentistry, replacement of teeth by means of dental implants has become a common treatment modality in recent years. Success of implant depends on various systemic and local factors near the implant. Due to these factors, implant failures may take place either as early failure which can be observed immediately postsurgical implant placement and its main reason is failure to establish proper osteointegration initially during wound healing period, while late failure can occur after occlusal loading. This was suggested due to breakdown of osteointegration occurred preloading. To avoid such unwanted outcomes a carful assessment of various factors that might contribute to the implant failure is crucial.

The present study is aimed to assess the factors influencing the survival of Straumann dental implants in Saudi population over a 3 three years period. A total of 484 dental implants were evaluated for identifying risk indicators by assessing several periodontal parameters.

In the present study PPD was significantly associated with presence of keratinized tissue width, and oral hygiene status. In relation to grafting and implant type, grafted sites showed significantly deeper PPD than non-grafted sites and bone level implants showed deeper PPD compared to tissue level implants. These results were in accordance with French et al. who found greater probing depths at grafted sites when compared with non-grafted areas^19^.

BOP was measured as to whether bleeding is present or absent to recognize presence of inflammation, dental implants however, tend to bleed more upon probing with less probing force when compared to natural tooth^20^. Positive BOP was observed in 42% of implants that were examined, which is in accordance with other previously published studies. French et al. used modified Sulcular bleeding index and found BOP is associated with mucositis^19^. In a similar study by Buser et al.^21^ using Mombelli’s bleeding index which reported that on mere punctuating, bleeding spots merely suggests injury to the perimplant supporting tissues and mombelli’s class 2 indicates mucositis, further they concluded that mucositis does not necessarily progress to peri-implantitis overtime^22^. Van velzan et al. using the same index as Buser et al. in the above-mentioned study, has found 20% of the implant sites showed BOP^23^.

Recently in a study carried by Stoker et al. BOP was reported in 14% of the sites examined^24^.

Costa FO et al. reported bleeding on peri-implant probing, periodontal probing depth, and the presence of periodontitis were associated with a higher risk of developing peri-implantitis^25^.

The BOP positive sites reported in this study are relatively higher when compared BOP reported in the previously published studies and this could be due to the difference in the index used to record the BOP. In the current study BOP was assessed as present or absent, whereas other published studies used more specific bleeding on probing indices.

One of the very interesting findings in this study was the impact the amount of keratinized tissue width around the dental implants, which was highlighted in many systematic reviews^26–29^. In the present study, sites with < 2 mm width of keratinized tissue showed significantly more edematous soft tissue when compared to those with > 2 mm KTW. These results are in accordance with Souza et al. who found implant sites with < 2 mm KTW showed more inflammation. There are several controversies regarding KTW around the implant and its importance, Wennstrom 2012^30^ and Esper 2012^31^ showed in their study that there was no major difference in the clinical parameters like plaque control, gingivitis, BOP and PPD as was seen in areas in with and without sufficient KTW.

Gunpinar et al. reported that when risk factors for periimplant disease were analyzed, in addition to PI and periodontitis, BOP, higher PPD and KTW width less than 2 mm were associated with occurrence of peri-implant mucositis and peri-implantitis^32^.

In the present study radiographic changes were assessed as having no change in bone level and threads exposed. Patients’ poor oral hygiene was found to be significantly associated with radiographic thread exposure. This might suggest that failure to control patient oral hygiene could be a risk factor contributing to future inflammation development around the implant and consequently implant failure.

Furthermore, the present study findings revealed that lack of a minimum of 2 mm of KTW was associated with radiographic implant thread exposure. Gunpinar et al. presumed that less KTW is linked to the plaque accumulation than the bone loss which is noticed around implants as the higher PI were scored in implants with KTW less than 2 mm^32^. Bengazi et al.^33^ in their study reported higher crestal resorption and apical soft tissue positioning of implants placed in areas with insufficient KTW. In a recent study Van Ekeren et al.^34^ reported significantly lower crestal bone change in bone-level implants placed in an initial keratinized tissue thicknesses of 2 mm or less. There is enough evidence to highlight the importance of placing dental implants with enough keratinized tissue to avoid future complications such as bone loss around the implant^30–36^.

When planning implant placement various factors such as oral hygiene, keratinized tissues, bone must be considered, overlooking these factors can have a negative effect on the long-term success of the implant as reported here.

Several limitations had been observed in present study which include mainly inherent limitations of retrospective studies and inconsistent results from using different indices and tools in the methodology. In addition, the lack of follow up due to the retrospective design can affect the observation of a true association between different studied variables and outcomes measured. Additional limitation can be explained by collecting the present sample from one institute which cannot be highly efficient to translate the present findings to whole society of patients with Straumann implants and also having a considerably overall moderate sample size. Furthermore, the exclusion of classical and well-known risk factors related to implant diseases.

## Conclusion

Within the limitation of this study, the data presented supports the previously published data that bone loss with exposing the implants threads was significantly associated with poor oral hygiene and it highlighted about the importance of controlling variables like oral hygiene status and keratinized tissue level which would help to have a good radiographic stability of the implants. Well controlled long-term prospective cohort studies are required to further assess factors affecting survival of Straumann implants.

## Data Availability

The datasets used and/or analysed during the current study are available from the corresponding author on reasonable request.

## Abbreviations

BOP: Bleeding on probing
PI: Plaque index
KTW: Keratinized tissue width
MGJ: Muco-gingival junction
PPD: Periodontal pocket depth
